# 2021 Acknowledgment of *mBio* Reviewers

**DOI:** 10.1128/mbio.03682-21

**Published:** 2021-12-21

**Authors:** Arturo Casadevall

**Affiliations:** a Johns Hopkins Bloomberg School of Public Health, Baltimore, Maryland, USA

## EDITORIAL

On behalf of the editors of *mBio*, I gratefully acknowledge the following individuals, who served as reviewers for the journal in 2021. Their time and effort in reviewing articles have been essential in ensuring the high quality of our publications, and their help is greatly appreciated.

Kjersti Aagaard
Bahaa Kenawy Abuel-Hussien Abdel-Salam
Allison Abendroth
Michael C. Abt
Mark D. Adams
Philip P. Adams
Evelien M. Adriaenssens
Anton Aebischer
Abram Aertsen
Hector C. Aguilar
Hector Aguilar-Carreno
Daniel Aguirre de Carcer
Gillian M. Air
Tuomas Aivelo
Caroline M. Ajo-Franklin
Gajender Aleti
Rebecca Alexander
Mojtaba Alimolaei
Matthew Aliota
Irving Coy Allen
Kyle R. Allison
Francis Alonzo
Hal Alper
Alfred Amambua-Ngwa
Amal Amer
David R. Andes
Raul Andino
Anuska V. Andjelkovic
Norma Andrews
Elliot J. Androphy
Chris Anthony
Rafael Araos
Nancie M. Archin
Catherine R. Armbruster
Chelsie Elizabeth Armbruster
Sassan Asgari
David S. Askew
Gemma C. Atkinson
Jonathon P. Audia
Lynn Htet Htet Aung
Frank O. Aylward
Joanna Azaredo
Herwig Bachmann
Gilad Bachrach
Steffen Backert
Sangsu Bae
Taeok Bae
Yang Bai
Goran Bajic
Scott E. Baker
Susan C. Baker
Siddharth Balachandran
Kithiganahalli Narayanaswamy Balaji
Megan Tierney Baldridge
Carlos Balsolobre
Gert Bange
Katharine J. Bar
Giovana Cappio Barazzone
Daniel Barber
Stefanie Barbirz
Bridget M. Barker
Melanie J. Barnett
Christian Baron
Francisco Barona-Gomez
Frederic J. Barras
Douglas H. Bartlett
Mirko Basen
Christopher F. Basler
Aurelia Battesti
Buzz Baum
Sebastian Baumgarten
Andreas J. Bäumler
Gwyn A. Beattie
Josh R. Beck
Eric D. Becraft
Jose A. Bengoechea
J. Philipp Benz
Mary Berbee
Stephen B. Beres
Bork A. Berghoff
Ben C. Berks
Thomas G. Bernhardt
Francesco Berti
Stefan Bertilsson
Sonja M. Best
Sébastien Besteiro
Carmen R. Beuzon
Steve Beverley
Gira Bhabha
Sumita Bhaduri-Mcintosh
Ashok S. Bhagwat
Jennifer F. Biddle
Dawn R. D. Bignell
Robert Blake Billmyre
Jordan E. Bisanz
Thomas Bjarnsholt
Mike Blackman
Daniel Blanco-Melo
Michael Blatzer
Mike Blazanin
James B. Bliska
Louis-Marie Bobay
Anamelia Lorenzetti Bocca
Jin Woo Bok
Yannick J. Bomble
Juan Bonachela
Matteo Bonazzi
Alisdair Boraston
Manuel V. Borca
Anthony Borneman
Helena Ingrid Boshoff
Alberto Bosque
Michael Bott
Fadila Bouamr
Sophie Bouillet
Nathalie Boulanger
Christina R. Bourne
Kostas Bourtzis
Eric Boyd
Jeffrey Michael Boyd
Günter Brader
Peter J. Bradley
Alessandra Bragonzi
Axel A. Brakhage
Marc Bramkamp
Jason M. Brenchley
Bruce James Brew
Ariane Briegel
Christopher John Brigham
Melinda Ann Brindley
Shaun R. Brinsmade
Catherine Brissette
Andrew J. Broadbent
Inka Brockhausen
Michael Brockhurst
Mark A. Brockman
Igor E. Brodsky
Barbara Bröker
Juliana Delatorre Bronzato
Christopher B. Brooke
Roland Brosch
Daren W. Brown
Jeremy S. Brown
Kevin M. Brown
Pamela J. B. Brown
John H. Brumell
Harry Brumer
Harald Brüssow
Randy Brutkiewicz
Michael J. Buchmeier
Amy H. Buck
Daniel H. Buckley
Angus Buckling
Lori L. Burrows
Stephen Busby
Carlos Andres Buscaglia
Alejandro Buschiazzo
Allen R. Buskirk
Geraldine Butler
Julea N. Butt
Mark J. Buttner
Mariana X. Byndloss
Sarah Louise Caddy
Eric Calvo
Andrew Camilli
David A. Campbell
Samuel K. Campos
Rafael Cantón
Adam Capoferri
Douglas G. Capone
Valerie Jean Carabetta
Nicholas H. Carbonetti
Jason A. Carlyon
Ronan K. Carroll
Vern B. Carruthers
Carol A. Carter
Dee A. Carter
Agostinho Carvalho
Leonardo J. M. Carvalho
Josep Casadesús
James E. Casanova
Eric Cascales
Jim Cassat
Roberto Cattaneo
Rick Cavicchioli
Gary Cecchini
Chang-Jun Cha
Georgios Chamilos
Patricia Ann Champion
Jasper F. W. Chan
Jeff H. Chang
Matthew Chang
Shin C. Chang
Yanjie (Jay) Chao
Subhadeep Chatterjee
Dhruba K. Chattoraj
Hualan Chen
Ling-Ling Chen
Yin Chen
Haiping Cheng
Liang Cheng
Yanfen Cheng
Anne Chevallereau
Peter Chien
Chetan E. Chitnis
Shu-Sin Chng
Byung-Kwan Cho
Hongbaek Cho
Anthony H. Choi
Young Bong Choi
Nicolas Chomont
Yit Heng Chooi
Mallory J. Choudoir
Nicholas P. Cianciotto
Jan Claesen
Antoine Claessens
Eric Clambey
Thomas A. Clarke
Anna R. Cliffe
Ian Cockburn
Tom Coenye
Gary H. Cohen
Randall J. Cohrs
Maureen L. Coleman
Maria Carmen Collado
Brian Conlon
David Cook
Laura Cook
Andrea Cooper
Isabelle Coppens
Brendan Cormack
Rebecca M. Corrigan
Joseph Cotruvo
Peggy A. Cotter
John V. Cox
Robert Craigie
Robert A. Cramer
Ileana M. Cristea
Robert W. Cross
Sean Crosson
Sean Andrew Crowe
Luis Cruz-Vera
Liwang Cui
Bryan R. Cullen
Paul J. Cullen
Valeria C. Culotta
Kyle W. Cunningham
Christina A. Cuomo
Cameron R. Currie
Mirjam Czjzek
Tal Dagan
Ya-Nan Dai
Tobias Dallenga
Andre Damasio
Ajai A. Dandekar
Scott G. Daniel
Pranav Danthi
Daniel Dar
F. Darfeuille
Richard Darveau
Andrew J. Darwin
Sandeep Kumar Dash
Alan R. Davidson
Andrew D. Davidson
Bryan W. Davies
Dana A. Davis
Ian Christopher Davis
Kimberly Michele Davis
Patricia Martine Day
Paul L. DeAngelis
Leandro José de Assis
Zeger Debyser
Luiz Pedro Sorio de Carvalho
Edward Deehan
Sandie Degnan
Marcus de Goffau
Christopher Luis de Graffenried
Sam Degregori
Kirk W. Deitsch
Robert Delatolla
Juan Carlos de la Torre
Nicholas R. De Lay
Frank R. DeLeo
Neal A. DeLuca
Carlos Maluquer de Motes
Marianne De Paepe
Jie Deng
Eric Y. Denkers
John J. Dennehy
Solène Denolly
William H. DePas
Keith M. Derbyshire
Cynthia Ann Derdeyn
Hilde De Reuse
Manu De Ryker
Stuti Desai
Albert Descoteaux
Ulrich Desselberger
Rik L. de Swart
Rory D. de Vries
Neelendu Dey
Eric Déziel
Lorena Díaz
George C. diCenzo
Thomas Dick
Allison Louise Didychuk
Diego G. Diel
Binh An Diep
Bruno Martorelli Di Genova
Stephen P. Diggle
Joseph P. Dillard
Ahmad Ud Din
Julia Dinasquet
Siyuan Ding
Marc S. Dionne
Meike Dittman
Elke Dittmann
Maziar Divangahi
Scott Dixon
Ulrich Dobrindt
Tobias Doerr
Yohei Doi
Min Dong
Sarah E. F. D’Orazio
Patricia C. Dos Santos
Thomas J. Dougherty
Simon L. Dove
Michael Downey
Diana M. Downs
Charles M. Dozois
Marlene Dreux
Brian T. Driscoll
Rebecca Drummond
Grégory Dubourg
Breck A. Duerkop
Peter Duerre
Yves F. Dufrene
Guillaume Dumenil
Ildiko Rita Dunay
Katherine R. Duncan
Anne K. Dunn
Gary M. Dunny
Sylvain Durand
Gonzalo Durante-Rodríguez
Rachel J. Dutton
Jeffrey D. Dvorin
Jonathan Dworkin
Daniel Ebbole
Gregory D. Ebel
Leo Eberl
Hideki Ebihara
Anastassios Economou
Mira Edgerton
Martin Egan
Nasreen Zafar Ehtesham
Maryna C. Eichelberger
Patrick Eichenberger
Meri Eichner
Catherine Eichwald
Shokrollah Elahi
Eran Elinav
Marie A. Elliot
Joanne Engel
Pablo Engel
Philipp Engel
David M. Engelthaler
David M. Engman
Hyungjin Eoh
Marc Erhardt
Kimberly D. Erickson
Joel Ernst
Jorge C. Escalante-Semerena
Rosa Esteban
Prahathees Eswara
Eliseo A. Eugenin
Ana Eulalio
Franziska Faber
Hillmann Falk
Ann Fallon
Gang Fang
Weijia Fang
Rhys Farrer
Michael J. Federle
Anthony R. Fehr
Mario F. Feldman
Pinghui Feng
Youzhi Feng
Jessie Fernandez
Ana Fernandez-Sesma
Dominique Ferrandon
Sebastien Ferreira-Cerca
Paul D. Fey
Alain Filloux
Arnaud Firon
Matthew C. Fisher
Connor Fitzpatrick
Erik K. Flemington
Josué Flores-Kim
Paul Christopher Michael Fogg
Edan Foley
Anja Forche
Jarrod R. Fortwendel
Simon J. Foster
Timothy J. Foster
George E. Fox
Leigh A. Frame
Pilar Francino
Jean M. Francois
Achilleas Frangakis
Gad Frankel
David R. Franz
Kurt Frederick
David N. Fredricks
Eric O. Freed
Karine Frenal
Matthew B. Frieman
Shinji Fukuda
Klaus Fütterer
Adam Gaballe
Iwona Gabriel
Michael Gale
Tom Gallagher
Michael Y. Galperin
Beile Gao
Leonor García-Bayona
Mariano A. Garcia-Blanco
Francisco Garcia-Cozar
José Manuel García-Fernández
Ferran Garcia-Pichel
Adolfo García-Sastre
Andrew F. Gardner
Victoriano Garre
Robert F. Garry, Jr.
Beth A. Garvy
Peter Gaskill
Pananghat Gayathri
Baoxue Ge
Barney Geddes
Thomas Geisbert
Ivelin Georgiev
George Georgiou
Kenn Gerdes
William H. Gerwick
Jean-Marc Ghigo
Karine Gibbs
David P. Giedroc
Scott Michael Gifford
Francis Gigliotti
Harry Gilbert
Joseph James Gillespie
Fabienne Girard-Misguich
N. Louise Glass
Elizabeth Glennon
Erin S. Gloag
William E. Goldman
Erin D. Goley
Mark Gomelsky
Andrew L. Goodman
Nilu Goonetilleke
Friedrich Götz
Elena A. Govorkova
Marcin Grabowicz
Jeffrey A. Gralnick
Guido Grandi
Christophe Grangeasse
Michael Jeffrey Gray
David E. Greenberg
Alexander L. Greninger
David Gresham
Gilbert Greub
Jennifer T. Grier
Scott S. Grieshaber
Elisabeth Grohmann
Philippe Gros
Samantha Gruenheid
Angelika Gründling
Fabio Gsaller
April Gu
John Guatelli
Keith Gull
Haitao Guo
Ju-Tao Guo
Dinesh Gupta
Maria Hadjifrangiskou
Matthias Hahn
Rong Hai
Sven Halbedel
Benjamin G. Hale
Ruth M. Hall
Jessica L. Halpin
Kimberly H. Halsey
Trinity L. Hamilton
Ming Hammond
Lynn E. Hancock
Meaghan H. Hancock
William R. Harcombe
Tilmann Harder
Clare Harding
Alexander Harms
Steven D. Harris
Tajie Harris
Takao Hashiguchi
Hassan Hashimi
Tomohisa Hasunuma
Asma Hatoum-Aslan
Toshio Hattori
Tracy H. Hazen
Lian He
Aoife T. Heaslip
Julian Hegemann
David E. Heinrichs
Elmar Heinzle
Sophie Helaine
Hanneke Hemmink
Andrew Henderson
Jeffrey P. Henderson
David R. Hendrixson
Tory A. Hendry
Scott E. Hensley
Ute Hentschel
Satu Hepojoki
Joshua Herbeck
Jennifer K. Herman
Betsy Herold
Alfredo H. Herrera-Estrella
Antonia Herrero
Martina Herrmann
Alon Herschhorn
Volker Heussler
Penelope Higgs
Kent L. Hill
Russ Hille
Karsten Hiller
Suzanne Hingley-Wilson
Vanessa M. Hirsch
Chris Todd Hittinger
Brian T. Ho
Ya-Chi Ho
Antoine Hocher
Martin Hoenigl
Markus Hoffmann
Alexander R. Horswill
Nancy Horton
Paul A. Hoskisson
Edith N. G. Houben
Andrew J. Hryckowian
Michael H. Hsieh
Haitao Hu
Jianming Hu
Ke Hu
Wenhui Hu
Yao-Wei Huang
Stéphane Hué
Luciano F. Huergo
Diego Huet
Diarmaid Hughes
Deborah T. Hung
Christopher Hunter
Ryan C. Hunter
Wasim Hussain
Christopher Dwight Huston
Geelsu Hwang
Kevin Hybiske
Joseph M. Hyser
Michael Ibba
Ashraf Ibrahim
James Imlay
Danielle J. Ingle
Thomas J. Inzana
Thomas R. Ioerger
Ronald M. Iorio
Salim Islam
Nahed Ismail
Masahiro Ito
Satoko Iwahori
Bertram L. Jacobs
William R. Jacobs, Jr.
Allan S. Jacobson
Swati Jain
Ursula Jakob
Tim James
Eleanor Jameson
Guilhem Janbon
Monika Janczarek
Aude Jary
Babak Javid
Lee-Ann Jaykus
Greg Jedd
Vicki Jeffers
Grant J. Jensen
Patric Jern
Hongpeng Jia
Cong Jiang
Daohong Jiang
Lu-Bin Jiang
Meixiu Jiang
Shibo Jiang
Xiuping Jiang
Vladimir Jiranek
Michael Joannidis
Dirk Jochmans
Jörgen Johansson
Richard John
Pål Jarle Johnsen
Eachan Johnson
Marc C. Johnson
Clare Jolly
Clinton J. Jones
R. Brad Jones
Matthew A. Jorgenson
Olivier Julien
Vladimir R. Kaberdin
Betul Kacar
Barbara C. Kahl
Niilo Kaldalu
Jeremy Phillip Kamil
Melissa Kane
Theodoros Karapantsios
David Karig
John Karijolich
Andrey V. Karlyshev
Krishanpal Karmodiya
Fatah Kashanchi
Elena Nikolaevna Kashinskaya
Matthew T. Kasson
Laura A. Katz
Atsushi Kawaguchi
Yoshihiro Kawaoka
Paul Kaye
Barbara I. Kazmierczak
Hangjun Ke
Daniel B. Kearns
Thomas E. Kehl-Fie
Harald Kellner
John M. Kelly
Jack Kennell
Linda J. Kenney
Stephen J. Kent
Peter Keyel
Nemat O. Keyhani
M. Nadeem Khan
Noor Khan
Anupama Khare
Megan R. Kiedrowski
Laura Kiessling
Yoshitomo Kikuchi
Patricia J. Kiley
Baek Kim
Dae Hyuk Kim
Hyun Uk Kim
Paul R. Kinchington
Taroh Kinoshita
David L. Kirchman
Natalia V. Kirienko
Lucas Kirschman
Adrienne Kish
Kasper Urup Kjeldsen
Jonathan L. Klassen
Nichole R. Klatt
Colin Kleanthous
Eric A. Klein
Robyn S. Klein
Sabra L. Klein
Karl E. Klose
Leigh A. Knodler
Laura J. Knoll
Kazuo Kobayashi
Ryuichi Koga
Alan Koh
Jay Kolls
Roberto Kolter
Masaaki Komatsu
Anna Konavalova
James B. Konopka
Anna Konovalova
Kostas Konstantinidis
Daniel Kornitzer
Benoit Kornmann
Nicole Marie Koropatkin
Britt Koskella
Saori Kosono
Joel E. Kostka
Erika Kothe
Vassili N. Kouvelis
Atsushi Kouzuma
Ákos T. Kovács
Pavel Kovarik
Thomas R. Kozel
Anne Marie Krachler
Joel Kralj
Florian Krammer
Philip Kransuzch
Philip R. Krause
Bernhard Krautler
Barry N. Kreiswirth
Tino Krell
Jens Kreth
Christopher J. Kristich
Lee Kroos
Mart Krupovic
Damian J. Krysan
Yasuyuki Kubo
Karl Kuchler
Meta J. Kuehn
Ursula Kües
Richard J. Kuhn
Ayush Kumar
Muthiah Kumaraswami
Mark Kuniholm
Oliver Kurzai
Jason Kwan
Kyung J. Kwon-Chung
Isabelle Laforest-Lapointe
Michael M. C. Lai
Rebecca Lamason
Paul F. Lambert
Louis Lambrecht
Peter Lammers
Nathaniel Roy Landau
Stephanie Langel
Ryan A. Langlois
Gordon Langsley
Johan Larsbrink
Inigo Lasa
Amel Latifi
Rob Lavigne
Helen M. Lazear
Tung Le
Maryse LeBrun
Benhur Lee
Chia Y. Lee
Jean Claire Lee
Kyu-Ho Lee
Richard Lee
Seok-Yong Lee
Soo Chan Lee
Benfang Lei
Nicholas J. Lennemann
Jay T. Lennon
Sophie Lepoder
Karine LeRoch
Cammie F. Lesser
Petra Anne Levin
Tera Levin
Stuart M. Levitz
Shoshana Levy
Kim Lewis
Zachary A. Lewis
Ruth E. Ley
Bibo Li
Jianrong Li
Kui Li
Meng Li
Renfeng Li
Sean Li
Shitao Li
Yize Li
Alexander Lichius
Fang Yun Lim
Jingjun Lin
Tao Lin
Scott E. Lindner
Steven E. Lindow
W. Ian Lipkin
John J. LiPuma
Trevor Lithgow
Yael Litvak
Guanqun Liu
Huaiwei Liu
Jinhua Liu
Shan-Lu Liu
Wei Liu
Wende Liu
Per O. Ljungdahl
Shawn R. Lockhart
Olga Lomovskaya
Sarah Londrigan
Hongan Long
Carolina B. Lopez
Remy Loris
Hannelore Lotter
Flávio V. Loures
Andrew Lee Lovering
Tiffany M. Lowe-Power
Pierre-Yves Lozach
Guodong Lu
Ling Lu
Yang Lu
Jun-Bo Luan
Jeremy Luban
William Ludington
Micah A. Luftig
Joen Luirink
Ben F. Luisi
Tania Lupoli
Joseph Lutkenhaus
Frank Lyko
Amy T. Ma
Bing Ma
Feng Ma
Kevin R. Macaluso
Iain MacArthur
Duncan R. MacCannell
Frank Madeo
Goedele Maertens
Paul Magwene
Bernardo Alfredo Mainou
Shinji Makino
Fabienne Malagnac
Frank Maldarelli
Jacob Malone
Reet Mändar
Mark J. Mandel
Alexander Mankin
Ivan Marazzi
Henju Marjuki
Luciano A. Marraffini
Benoît S. Marteyn
José Luis Martínez
Luis Martínez-Sobrido
Vincent G. Martinson
Eric Massé
Florent Masson
Amy J. Mathers
Greg Matlashewski
Yasufumi Matsumura
Yoshiharu Matsuura
Yu Matsuura
Keith R. Matthews
Daniel R. Matute
Wendy Maury
Anthony Maxwell
Meghan May
Xavier Mayali
Didier Mazel
Divingahi Mazier
Jere W. McBride
Grant McClarty
Malcolm J. McConville
Beth A. McCormick
John P. McCutcheon
Timothy R. McDermott
Sarah Marie McDonald Esstman
Quinn S. McFrederick
Maureen A. McGargill
Martin J. McGavin
Glenn McGregor
Michael J. McInerney
James B. McKinlay
Jeffrey Scott McLean
Jason S. McLellan
Jan R. Mead
Paul Mead
David G. Meckes
Supriya D. Mehta
Sahar Melamed
Jay Mellies
Didier Ménard
Frances Mercer
Jason Mercer
Timothy C. Meredith
Barbara Methe
Dennis W. Metzger
Justin Meyer
Craig Meyers
Paul Michels
Jan Michiels
Alita A. Miller
Kathryn C. Milligan-Myhre
Jonathan J. Miner
Aaron P. Mitchell
Amit Mitra
Shin-Ya Miyagishima
Jennifer F. Moffat
Andreas Möglich
Anne Moir
Isabella Moll
A. Mondragon
Andrew J. Monteith
Arnold Monto
Gary Peter Moran
Nancy A. Moran
Xosé Anxelu G. Morán
Karen Moreau
Hiroshi Mori
John Morissey
Renato Morona
Donald A. Morrison
John P. Morrissey
Joachim Morschhäuser
Tatum D. Mortimer
Bernard Moss
Paul Moss
Heba H. Mostafa
Maria Manuel Mota
Jarrod J. Mousa
Isabelle Mouyna
Patrick Moynihan
Monica Mugnier
Aindrila Mukhopadhyay
Volker Müller
Conrad W. Mullineaux
Joshua Munger
Karl Münger
Christian Münz
Alise Muok
Shin Murakami
Vasant Muralidharan
Eain A. Murphy
Timothy F. Murphy
Colin Murrell
James M. Musser
Peter J. Myler
Hannu Myllykallio
Hiroki Nagai
Ravinder Nagpal
László Nagy
Moon H. Nahm
Satish K. Nair
Yuji Naito
Nobutaka Nakashima
Sukanya Narasimhan
Franz Narberhaus
Janaína De Freitas Nascimento
William Wiley Navarre
Dipti D. Nayak
David M. Needham
Matthew Neiditch
Stuart J. Neil
Jeniel E. Nett
Michael M. Nevels
Hayley Joy Newton
Irene L. G. Newton
Olivier Neyrolles
Timothy J. Nice
John M. Nicholls
Francisco Esteban Nicolas
Carolyn Nielsen
Michael Niepmann
Nikolay E. Nifantiev
Pablo Ivan Nikel
Ken-Ichi Nishiyama
Justin R. Nodwell
Laura Mary Nolan
Philip J. Norris
Elizabeth B. Norton
Mairi C. Noverr
Mari Numata
Patricia Nuttall
Joshua J. Obar
Rebecca Oberley-Deegan
Megan A. O’Connor
Tamara O’Connor
Greg Odorizzi
David O’Dwyer
Stefan H. Oehlers
Molly Ohainle
Colm O’Huigin
Shinichi Oide
Hiroaki Okamoto
Ely Oliveira-Garcia
Michal A. Olszewski
Teresa R. O’Meara
Anders Omsland
Ángel Oñate
Michiel Op De Beeck
Robert C. Orchard
David A. Ornelles
William D. Orsi
Heinz D. Osiewacz
Sreekumar Othumpangat
George O’Toole
Karen M. Ottemann
F. Wayne Outten
Songying Ouyang
Christos A. Ouzounis
Will Overholt
Slobodan Paessler
Ranajit Pal
Bernhard O. Palsson
Eric Pamer
Hongyu Pan
Surya Prakash Pandey
Galina Pankratova
Guido Pappa
Alexander R. Paredez
Kristin N. Parent
Pyong Woo Park
John S. Parkinson
Griffith D. Parks
Andrew C. Pawlowski
Shelley M. Payne
Simone Pecetta
Virginia Pedicord
Joao Pedra
Richard M. Peek
Mark E. Peeples
Andrew Pekosz
José Penades
Miguel A. Penalva
Xu Peng
Ines C. Pereira
Daniel R. Perez
Gabriel G. Perron
Andreas Peschel
William A. Petri
Christian Karl Pfaller
Julie K. Pfeiffer
Martin Picard
Laura Piccio
Ted C. Pierson
Giampiero Pietrocola
Shiv Pillai
Paul J. Planet
Richard K. Plemper
Mircea Podar
Eric M. Poeschla
Stefan H. Pöhlmann
Norbert Polacek
Alessandra Polissi
Justin Pollara
Alexander Poltorak
Mauricio H. Pontes
David L. Popham
Matteo Porotto
Daniel A. Portnoy
Christopher Power
Rajendra Prasad
Andrew Preston
Jeffrey W. Priest
Sean T. Prigge
Alice S. Prince
Arthur Prindle
Hans Christian Probst
Jonathan Pruneda
Read Pukkila-Worley
John G. Purdy
Michael A. Purdy
Cheng-Feng Qin
Yongli Qin
Jianming Qiu
Joseph Qualls
Joshua Quick
Janet Quinn
Nancy Raab-Traub
Simona Radutoiu
Manuela Raffatellu
Paul B. Rainey
Tracy Lyn Raivio
Stuart A. Ralph
Maria Soledad Ramirez
Tariq Rana
Chad A. Rappleye
Philip N. Rather
Isabella Rauch
Kasie Raymann
Patrick C. Reading
Johannes G. Rebelein
Peter Redder
Matthew Reeves
E. Hesper Rego
Han Remaut
Michelle Lynne Reniere
Christopher Rensing
Yolanda Revilla
Nicole Reynolds
Todd B. Reynolds
Andrew Rice
Stephen A. Rice
Christopher D. Richardson
Jonathan P. Richardson
Douglas D. Richman
Justin M. Richner
Douglas Risser
Simon K.-M. R. Rittman
Rafael Rivilla
Dwayne Roach
Nadia Roan
Richard J. Roberts
Derrick R. Robinson
Keven Mara Robinson
Michael Roche
Marcio L. Rodrigues
Andre Rodriguez
Davinia Salvachua Rodriguez
Luis Rodríguez-Rojas
Paul D. Roepe
Stephen J. Rogerson
Michael Roggendorf
Holger Rohde
Philippe Roingeard
Casey E. Romanoski
Tony Romeo
Diego Romero
Francesca Ronchi
Anthony Rongvaux
German Rosas-Acosta
Brad R. Rosenberg
Benjamin Ross
Stefan Rothenburg
Michael Rother
Andrew Routh
Francoise Helene Routier
Annette Rowe
Deodutta Roy
Gloria Rudenko
Christopher Ruis
Natividad Ruiz
Marta Ruiz-Riol
Françoise Rul
Kendra P. Rumbaugh
Tomáš Ruml
Jacob A. Russell
Michael W. Russell
Robert Sabatini
Matthew S. Sachs
David L. Sacks
Ben Sadd
Mohsan Saeed
James Peter Saenz
Saveez Saffarian
David Safronetz
H.-G. Sahl
Olga Sakwinska
Michaela M. Salcher
Hassan Salem
Stephen Salipante
Harini Sampath
Buka Samten
James E. Samuel
Laura M. Sanchez
Maria Sandkvist
Rozanne M. Sandri-Goldin
Hélène Sanfaçon
Dominique Sanglard
Álvaro San Millan
Thomas James Santangelo
Martin Sapp
Ligia M. Saraiva
Antonia Sargona
L. Peter Sarin
Ilker Kudret Sariyer
Saumendra N. Sarkar
Peter Sarnow
Varun Sasidharan
Kei Sato
Yorifumi Satou
Yoko Satta
Quentin J. Sattentau
David F. Savage
Stephen P. Saville
Joy Scaria
Matthew M. Schaefers
Luis M. Schang
Dirk-Jan Scheffers
Stefan Schild
Martin Schlee
Susan Schlimpert
Mirco Schmolke
Monika Schmoll
Bernd Schnabl
Dirk Schneider
Juergen Schneider-Schaules
Michael Schotsaert
Tony Schountz
Klaus Schughart
Olivier Schwartz
Julia A. Schwartzman
Herbert P. Schweizer
Martin Schwemmle
Jessica Scoffield
Barry Scott
Patrick R. Secor
Florian Seebeck
Frank Seeber
Anna Maria Seekatz
Meredith Seeley
Kate L. Seib
Ryan F. Seipke
Maria Selmer
Alecia N. Septer
Laura R. Serbus
Joshua Shaevitz
William M. Shafer
Alon Shaiber
Reut Shalgi
Stephanie R. Shames
Feng Shao
Amir Sharon
Thomas J. Sharpton
Karen Joy Shaw
Lindsey N. Shaw
Megan L. Shaw
Timothy P. Sheahan
Ji-long Shen
Pei-Yong Shi
Yi Shi
Zheng-Li Shi
Zhongjie Shi
Nicholas J. Shikuma
Kwang-Soo Shin
Masaki Shintani
Erika Shor
Joshua D. Shrout
Jessica R. Sieber
Alex Sigal
Paul Sigala
Ariel Mariano Silber
Robert F. Siliciano
Lynn L. Silver
Gregg J. Silverman
Patricia J. Simner
Augusto Simoes-Barbosa
David A. Six
Rebecca L. Skalsky
Mikael Skurnik
James M. Slauch
Nicolas Sluis-Cremer
Mark S. Smeltzer
James R. Smiley
Despina Smirlis
Jessica A. Smith
Joseph D. Smith
Terry K. Smith
Eveline Snelders
Jonathan W. Snow
Hokyoung Son
Fernando C. Soncini
Holger Sondermann
Hao Song
Harini Sooryanarain
Joseph A. Sorg
Kirsten Spann
Paul Spearman
Charles Specht
Tobias Spielmann
Katherine R. Spindler
Alfred Michael Spormann
Eric V. Stabb
Jason E. Stajich
Thomas Stamminger
Nicola R. Stanley-Wall
Claude (Chad) Steele
James C. Stegen
Joan A. Steitz
Silke Stertz
Ann M. Stevens
Brian Stevenson
Timothy P. Stinear
Ann M. Stock
John F. Stolz
Stefano Stracquadanio
Henrik Strahl
Daniel N. Streblow
Matt Stremlau
Ashley Strickland
Giannis Stringlis
Natalie C. J. Strynadka
Jorg Stulke
Wei Wen Su
Mrutyunjay Suar
Selvakumar Subbian
Motoyuki Sugai
William Sullivan
Paul Sumby
Jingru Sun
Weina Sun
George W. Sundin
Mehul S. Suthar
Mark D. Sutton
Richard E. Sutton
Nobuhiro Suzuki
Marc Swidergall
W. Edward Swords
Makoto Takeda
Norio Takeshita
Demeng Tan
Sen-Lin Tang
Yinjie J. Tang
Azuma Taoka
Rick L. Tarleton
Stefan Taube
John E. Tavis
Meredith Teilhet
Luis Teixeira
Olivier Tenaillon
Scott S. Terhune
Herve Tettelin
Bas Teusink
Aartjan J. W. te Velthuis
Rita Tewari
Christopher Thibodeaux
Robert Thimme
Gavin H. Thomas
Paul G. Thomas
Linda S. Thomashow
Bart P. H. J. Thomma
Luke R. Thompson
J. Cameron Thrash
Chaoguang Tian
Mariana Tinajero-Trejo
Aaron A. R. Tobian
Kenzo Tokunaga
Nobuhiko Tokuriki
Martin Tolstrup
M. Alejandra Tortorici
Karoly Toth
Frances Trail
Paula Traktman
Satyajit Tripathy
Jerry M. Troutman
Yuk-Ching Tse-Dinh
Paola Turano
Kürsad Turgay
Peter J. Turnbaugh
Keith Turner
Wendy W. J. Unger
Rajendra Upadhya
Shuzo Urata
Akhil B. Vaidya
Raphael Valdivia
John Valentine
Martina Valentini
Maria Valeri
Tenaya Vallery
Bert van den Berg
Bram Van den Bergh
Marjan W. van der Woude
Patrick Van Dijck
Giel G. van Dooren
Julia C. van Kessel
Carine Van Lint
Laurence Van Melderen
Debby van Riel
Willem van Schaik
Gilles P. van Wezel
Vladimir Varga
Julien Vaubourgeix
Andres Vazquez-Torres
Joel Vega-Rodríguez
Jonathan L. Vennerstrom
Stephanus N. Venter
Wim F. J. Vermaas
Gabriela Nogueira Viçosa
Anastasia N. Vlasova
Waldemar Vollmer
Dorothee von Laer
Christoph Vorburger
Julia A. Vorholt
Daniel E. Voth
Andreas Wack
Joseph Thomas Wade
Aljoscha S. Wahl
Jacob R. Waldbauer
Matthew K. Waldor
Kevin John Waldron
Seth T. Walk
David H. Walker
Jennifer Walker
Nicholas A. Wallace
Maroya S. Walters
Paul Walther
Jianjun Wang
Jianwei Wang
Jincheng Wang
Leyi Wang
Linqi Wang
Qiuhong Wang
Sibao Wang
Taia T. Wang
Xiaoxue Wang
Yanbo Wang
Yue Wang
Matthew J. Wargo
Digby F. Warner
Martin J. Warren
Andrew P. Waters
Christopher M. Waters
Lauren Waters
Robert O. Watson
Kylie J. Watts
Scott C. Weaver
Gunter Wegener
James Weger-Lucarelli
Aaron Weimann
Deborah J. Weis
Jeffrey N. Weiser
Carol D. Weiss
Howie Weiss
David Welch
Rory M. Welsh
Zezhang Tom Wen
Tim Wencewicz
A. Phillip West
Edze Westra
I. Wheeldon
Richard Wheeler
Sean P. J. Whelan
Michael White
Aaron Whiteley
Katrine Whiteson
Craig B. Wilen
Claus O. Wilke
Robert J. Wilkinson
Rob J. L. Willems
Anusuya Willis
Alex Wilson
Duncan W. Wilson
Michael R. Wilson
Richard A. Wilson
Sebastian E. Winter
Genevieve L. Wojcik
Alan J. Wolfe
Kenneth H. Wolfe
Christiane Wolz
Richard Wong
Daniel J. Wozniak
Karen Wozniak
Rachel A. F. Wozniak
Brendan W. Wren
Antoni Wrobel
Weihui Wu
Zhiqiang Wu
Ning-Shao Xia
Xin Xiang
Yan Xiang
Yan Q. Xiong
Min Xu
Chaoyang Xue
Gilad Yakov
Masahiro Yamamoto
Seiya Yamayoshi
Qingyun Yan
Ching-Hong Yang
Ence Yang
Liang Yang
George S. Yap
Ellen Yeh
Hui-Ling Yen
Kristine Yoder
Hyunah Yoon
Jae-Hyuk Yu
Qigui Yu
Yunsong Yu
Jing Yuan
Joshua S. Yuan
Lijuan Yuan
Cheol-Won Yun
Joseph P. Zackular
Muhammad Ammar Zafar
Patricia Zambriski
David Zamorano-Sánchez
Egija Zaura
Jonathan Zehr
Quan Zeng
Bing Zhai
Dabing Zhang
Jianqiang Zhang
Jie Zhang
Junfeng Zhang
Wei Zhang
Xiaojie Zhang
Zhengguang Zhang
Bo Zhao
Jincun Zhao
Youbao Zhao
Shijun J. Zheng
Jin Zhou
Pei Zhou
Tongqing Zhou
Jian Zhu
Guangchao Zhuang
John Ziebuhr
Dan Zilberstein
Sadri Znaidi
Susan B. Zolla-Pazner
John Zupan
Daniel Vincent Zurawski

